# One-fourth of the prisoners are underweight in Northern Ethiopia: a cross-sectional study

**DOI:** 10.1186/s12889-017-4410-9

**Published:** 2017-05-15

**Authors:** Semaw Ferede Abera, Kelemework Adane

**Affiliations:** 1 School of Public Health, College of Health Sciences, Mekelle University, Mekelle, Ethiopia; 2 Kilte Awlaelo- Health and Demographic Surveillance Site, Mekelle University, Tigray, Ethiopia; 3 Department of Medical Microbiology and Immunology, College of Health Sciences, Mekelle University, Mekelle, Ethiopia

## Abstract

**Background:**

Despite the fact that prisoners are exposed to different health problems, prison health problems are often overlooked by researchers and no previous study has investigated nutritional problems of prisoners in Ethiopia.

**Methods:**

Cross-sectional data were collected from 809 prisoners from nine major prison setups in the Tigray region of Ethiopia. A proportional stratified sampling technique was used to select the total number of participants needed from each prison site. The outcome of this study was underweight defined as body mass index (BMI) of less than 18.5 kg/m^2^. Multivariable binary logistic regression was performed to identify determinants of underweight at a *p*-value of less than 0.05.

**Results:**

The prevalence of underweight was 25.2% (95% CI; 22.3%- 28.3%). Khat Chewing (OR = 2.08; 95% CI = 1.17, 3.70) and longer duration of incarceration (OR = 1.07; 95% CI = 1.01, 1.14) were associated with a significantly increased risk of underweight. Additionally, previous incarceration (OR = 1.54; 95% CI = 0.99, 2.42) was a relevant determinant of underweight with a borderline significance. In contrast, family support (OR = 0.61; 95% CI = 0.43, 0.85) and farmer occupation (OR = 0.59; 95% CI = 0.36, 0.98) compared to those who were unemployed were important protective determinants significantly associated with lower risk of underweight.

**Conclusion:**

In summary, the burden of underweight was higher among prisoners in Tigray region who had respiratory tract infections. The study has enhanced our understanding of the determinants of underweight in the prison population. We strongly recommend that nutritional ﻿support﻿, such as therapeutic feeding programs for severely or moderately underweight prisoners, and environmental health interventions of the prison setups should be urgently implemented to correct the uncovered nutritional problem and its associated factors for improving the health status of prisoners.

## Background

Prisoners are exposed to a number of unfavorable health and health deterring factors including higher risks of mortality and injuries [1–5]. A remarkable feature of prisoners is that they often arrive at health service centers with a number of health and social problems which can lead to a high risk of malnutrition [6–8].

The literature indicates that prisoners face different nutritional problems ranging from several micronutrient deficiencies, re-emerging severe diet related diseases to delayed recovery, mental illness, sexual health problems, infectious diseases, a wide range of risk factors and increased risk of mortality [6, 7, 9–20].

Studies reported that poor nutrition was significantly associated with severity of pneumococcal pneumonia and risk of acquiring tuberculosis infection [21, 22]. Several other studies showed that underweight and micronutrient deficiencies were associated with higher odds of respiratory infections [23–25].

In general, in Africa, there is limited evidence on the various health problems of prisoners despite the relevance of such evidence on the health of the prisoners, their inmates, and the general community. In the case of Ethiopia, the prison health system seems to be not well integrated with the national health system and health problems of prisoners are mostly marginalized by researchers.

According to the 2015 report by World Prison Brief (WPB) and Institute for Criminal Policy Research (ICPR), in Ethiopia, the total prison population has grown by 66.9% since 2000, reaching 104,467 in 2010/2011 [26]. One of the basic human rights that prisoners, according to the Universal Declaration of Human Rights (UDHR) Article 25 (1), must have is access to adequate and healthy food choices [27] to meet their nutritional needs. Additionally, Mandela rule 22 states that “Every prisoner shall be provided by the prison administration at the usual hours with food of nutritional value adequate for health and strength, of wholesome quality and well prepared and served. Drinking water shall be available to every prisoner whenever he or she needs it.” [28].

To the authors’ best knowledge, the prevalence and associated factors of underweight has been scarcely researched from the perspective of prison settings. Therefore, this paper determines the prevalence and determinants of underweight among adult prisoners who had respiratory tract infections from 6 zonal and 3 district prison institutions located in Tigray region, northern Ethiopia.

## Methods

### Study area, setting and period

This study was implemented in Tigray region in northern Ethiopia as part of the tuberculosis (TB) study in prisons [29]. In the study, it was reported that the prevalence of culture confirmed TB was 4% (95% CI: 2.65–5.35).

In the regional state, there are nine major zonal and district prison institutions. During our study, there were 2557 prisoners in Mekelle prison site, 1513 in Shire, 1093 in Adigrat, 634 in Wukro, 653 in Axum, 537 in Adwa, 786 in Maichew, 710 in Alamata, and 875 in Humera. A quantitative cross sectional study design was conducted in all these nine prison sites with a source population of 9326 prisoners from August 2013 to February 2014.

### Inclusion and exclusion criteria

The inclusion criteria of our study were cough lasting for more than two weeks and fulfillment of at least one suggestive symptom of TB (such as night sweating, chest pain and/ or weight loss) among prisoners aged 18 years and above. Prisoners who fulfilled the inclusion criteria but were assessed to be mentally ill were excluded from the study.

### Source population, sample size calculation and sampling method

Of the total 9, 326 adult prisoners in the regional state, data were collected from 809 participants. Assuming a 50% prevalence of underweight, 5% precision, a design effect of 2, 95% confidence level and a 5% non-response rate, we calculated a sample size of 809 adult prisoners using a single proportion formula.

$$ \mathrm{n}=\frac{{\mathrm{z}}^2\mathrm{p}\left(1-\mathrm{p}\right)}{{\mathrm{d}}^2}=\frac{(1.96)^2(0.5)\left(1-05\right)}{(0.05)^2}=384.2\approx 385 $$

Accounting for the design effect of 2 and 5% expected response rate, the final sample size was 809. n_final_ = (385 X 2) = 770 + 5% non-response rate = 770 + 39 = 809 participants. Then, we multiplied the total prisoners in each of the nine prison sites by the strata-specific coefficient, 0.2432, to obtain the total number of prisoners from each prison site. Through room to room visits, with the guidance of the prison nurses in each site, data collectors asked if there were prisoners who suffered from coughing lasting two weeks or more. Next, we used a convenient sampling technique to select the eligible participants, who fulfilled the set inclusion criteria, from the total prisoners in each prison site. Finally, information about the objective and relevance of the study including detail guidance on informed consent was provided according to our study guidelines. Accordingly, selected participants in our study ranged from 46 in Adwa prison site to 222 in Mekelle prison site.

### Data collection and management

Experienced nurses and clinical officers screened the potential participants based on the inclusion criteria we set. Aiming to minimize inter-observer variation amongst our data collectors, training was given to the data collectors before they collected data using the structured questionnaire. Collected data from the questionnaire were double checked for completeness both by supervisors and data collectors. Accordingly, incomplete and implausible values were corrected on the spot.

### Outcome variable

The outcome of interest was underweight as measured using the body mass index (BMI), defined as the weight in kilograms divided by the square of the height in metres (kg/m^2^). The body weight was determined to the nearest 0.1 kg on a standing electronic digital scale and height was measured to the nearest 0.1 centimeter (cm). BMI was calculated for each participant and their nutritional status was categorized according to World Health Organization (WHO) standards [30, 31]. Accordingly, a participant was declared to be underweight if the BMI metrics is less than 18.5 kg/m^2^.

### Explanatory variables

Data on socio-demographic characteristics (age, sex, residence, marital status, education and occupation), family support, prison site, condition of the prisoners (whether the sentenced was or not), lifestyle profile during the study period (smoking, khat chewing) and before imprisonment (alcohol consumption, khat chewing) were collected. In addition, Human Immunodeficiency Virus (HIV) serological status, TB status, duration of cough, history and frequency of previous imprisonment and duration of current imprisonment were also obtained. HIV (1 + 2) Antibody Colloidal Gold (KHB) and Stat-Pak were used to initially test our samples and confirm the positive ones respectively. HIV-1/2 Unigold Recombinant assay was applied as a gold standard tie breaker for discordant HIV test results. Individual based pre-test and post-test HIV Counseling and Testing (HCT) was implemented confidentially according to the national guideline for HCT recommended by the Federal Ministry of Health (FMoH) of Ethiopia. Our diagnosis for pulmonary tuberculosis (PTB) was based on three consecutive early morning sputum samples. A bacteriologically confirmed positive PTB status was defined as a positive culture for *M.tuberculosis* and/or two smear positive sputum samples for the presumptive TB cases under a direct microscopy. Moreover, family support was operationally defined as family visit plus food aid or family visit plus money support or a combination of family visit, food aid and money support. Data on all other explanatory variables were taken from observation, such as sex of participants or from the response of the participants to each question during the interview.

### Statistical methods

The data was entered using Epi Data entry version 3.1 software. The raw dataset was exported to stata 13.0 for cleaning and data analysis. Frequency analysis was run to explore the range of values, identify missing data or possibly miscoded data. Then, a deep cautious exploratory data analysis was performed for the distribution of each continuous variable to prepare it for final analysis. Age and frequency of current imprisonment were not normally distributed and median and Interquartile range was used to summarize these variables.

Univariate association between each individual explanatory variable and underweight was evaluated using odds ratio (OR) with the corresponding 95% confidence interval (CI) as a parameter estimate. The next step was to build a statistical model, excluding the 22 overweight or obese cases, which identified the determinants of underweight among the adult prisoners. To build this model, all the predictor variables with a *p*-value of <0.05 on the bivariate analysis were fitted.

Accordingly, this model was built using age, family support, current smoking, current khat chewing and history of previous incarceration. In the next step, we purposely included additional explanatory variables which were statistically significant a *p*-value of <0.25 to build an empirical model [32]. In our analysis, a potential problem of multicollinearity was checked using variance inflation factor (VIF) at cut off point of 10 [33]. Interaction was also checked and we found no significant interaction terms. Finally, in the multivariable binary logistic model, a *p*-value of <0.05 was considered to declare statistical significance with the corresponding 95% confidence interval. The goodness of fit (GOF) of our model was checked using Hosmer-Lemeshow GOF test. The *p*-value of the Hosmer-Lemeshow GOF test of our model is 0.427 which confirms that the model is correctly specified.

## Results

### Overall situation of the prison setups

The largest prison institution in Tigray regional state is Mekelle prison, located in the state capital. During our observation, most prisons had very narrow windows while others had no windows at all. According to prison authority officials, the main reason of this was the fear that prisoners could escape. We also found that most prisoners had clinics which were poorly equipped, mostly with basic emergency level medical supplies, staffed by diploma holding nurses who were formerly assistant military nurses. There were no physicians or health professionals holding a Bachelor’s degree in any of the clinics. All the nurses reported that they did not participate in any formal training either by the regional health system or non-governmental organization.

From the perspective of the prisoners, in all the prison setups, prisoners complained of poor referral systems, bad environmental sanitation such as unsanitary toilets, problems of insomnia due to overcrowding and pests like fleas. Most study participants reported different dermatological problems such as itching. Additionally, 770 (95.2%), participants slept on the floor using a mattress mostly filled with grass or solely on carpet.

### Nutritional status of prisoners by socio-demographic characteristics

**Fig. 1:**
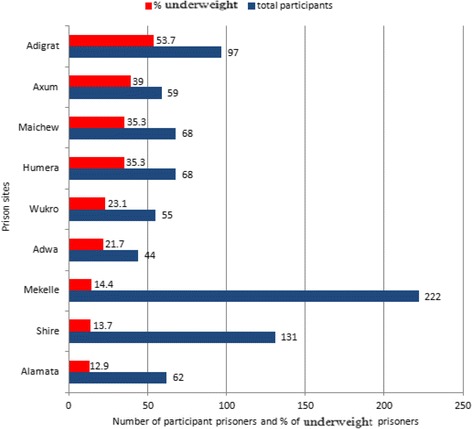
Total number of prisoners who participated in the study with the corresponding proportion of underweight prisoners by prison site in Tigray region, Ethiopia

The response rate was 100%. The prevalence of underweight among prisoners with respiratory tract infection was 25.2% (95% CI: 22.3%- 28.3%). The burden of underweight varies substantially from prison to prison, ranging from a minimum prevalence of 12.9% in Alamata prison site to a maximum of 53.7% in Adigrat prison site. Next to Adigrat, prisoners from Axum, Humera and Maichew prison sites had greater prevalence of underweight (Fig. 1). In our study, 29 (3.6%), 44 (5.4%) and131 (16.2%) of the prisoners were severely, moderately and mildly underweight respectively.

More than three-fifths, 124 (60.8%), of the underweight burden was among the age group 18-30. The mean age of those who were underweight was lower than those who were not underweight (Table 1).

**Table 1:** Nutritional status of prisoners by socio-demographic characteristics of adult prisoners in Tigray region, northern Ethiopia (*n* = 809)

Variables	Nutritional status		Total (*N* = 809)	*p*-value
	Not underweight, *n* (%); (*n* = 605)	Underweight, *n* (%); (*n* = 204)		
Age (median ± IQR)	30 ± 16	27 ± 16	29 ± 16	
Sex				*0.880*
Female	16 (76.2)	5 (23.8)	21	
Male	589 (74.8)	199 (25.3)	788	
Residence				*0.600*
Rural	339 (75.5)	110 (24.5)	449	
Urban	266 (73.9)	94 (26.1)	360	
Family support				0.001*
No	270 (64.4)	119 (30.6)	389	
Yes	335 (79.8)	85 (20.2)	420	
Marital status				*0.056*
Married	320 (77.7)	92 (22.3)	412	
Single	245 (70.6)	102 (29.4)	347	
Divorced/ widowed	40 (80)	10 (20)	50	
Educational status				*0.063*
No formal education	161 (77.4)	47 (22.6)	208	
Primary	290 (73)	107 (27)	397	
Secondary	109 (71.2)	44 (28.8)	153	
College and above	45 (88.2)	6 (11.8)	51	
Occupation				0.003*
Unemployed	86 (66.7)	43 (33.3)	129	
Civil servant	72 (78.3)	20 (21.7)	92	
Farmer	316 (79.6)	81 (20.4)	397	
Student	64 (64)	36 (36)	100	
Others occupation	67 (73.6)	24 (26.4)	91	

*indicates *p*-value <0.05 from the chi-square test analysis; *IQR * Inter Quartile Range

The prevalence of underweight was higher among prisoners who were not getting family support (30.6%), single by marital status (29.4%), and among people who had primary (27%) or secondary (28.8%) education. Moreover, those who were students (36%) and unemployed (33.3%) had also higher burden of underweight (Table 1).

### Nutritional status of prisoners by lifestyle, morbidity and other prison related conditions

The mean duration of incarceration was 29.7 months. Stratified by our outcome of interest, this was 28.8 months for those who were not underweight and 32.4 months for those who were underweight; this difference was statistically significant (*p* < 0.001). There was a 9.5% excess prevalence of underweight among prisoners who were smoking compared to those prisoners who were not smokers. Nearly half, 34 (44.7%), of those who chew khat were underweight compared to about one-fourth of those who did not chew khat. Furthermore, the prevalence of underweight was lower by 4% among prisoners who were imprisoned for less than 12 months compared to the prisoners who were incarcerated for longer than 12 months (Table 2).

**Table 2:** Lifestyle, morbidity and other prison related condition of adult prisoners in Tigray region, northern Ethiopia (*n* = 809)

Variables	Nutritional status		Total (*N* = 809)	*p*-value*
	Not underweight, *n* (%); (*n* = 605)	Underweight, *n* (%); (*n* = 204)		
Smoking status				0.011*
No	490 (76.8)	148 (23.2)	638	
Yes	115 (67.3)	56 (32.7)	171	
Chew khat				<0.001*
No	563 (76.8)	170 (23.2)	733	
Yes	42 (55.3)	34 (44.7)	76	
Alcohol use before jail				0.626
No	359 (74.2)	125 (25.8)	484	
Yes	246 (75.7)	79 (24.3)	325	
Duration of jail				0.203
< 12 months	250 (77.2)	74 (22.8)	324	
≥ 12 months	355 (73.2)	130 (26.8)	485	
HIV serostatus				0.251
Negative	581 (75.2)	192 (24.8)	773	
Positive	24 (66.7)	12 (33.3)	36	
Tuberculosis				0.103
No	585 (75.3)	192 (24.7)	777	
Yes	20 (62.5)	12 (37.5)	32	
Previous jail history				0.004*
No	520 (76.1)	163 (23.9)	683	
Yes	71 (63.4)	41 (36.6)	112	
Cough duration				0.690
2-4 weeks	216 (74)	76 (26)	292	
≥ 4 weeks	389 (75.2)	128 (24.8)	517	

* indicates *p*-value <0.05 from the chi-square test analysis

On the other hand, the prevalence of underweight was higher among prisoners who were imprisoned previously (12.7% excess prevalence), HIV positive (8.5% excess prevalence), and for those with culture confirmed PTB cases (12.8% excess prevalence) (Table 2). We also observed that the mean age of participants with a history of incarceration (25.4 years) was lower than the mean age of those who did not have a history of incarceration (30.3 years), but this difference was not statistically significant (*p* = 0.073).

### Factors associated with underweight among prisoners with respiratory infections

Adjusted for the fitted covariates shown in Table 3, prisoners who were supported by their families had a 39% (OR = 0.61; 95% CI = 0.43, 0.85) lower odds of underweight compared to those prisoners who were not getting family support. Another remarkable finding was that prisoners who reported chewing khat were two times (OR = 2.08; 95% CI = 1.17, 3.70) more likely to be underweight compared to those who did not chew khat (Table 3).

**Table 3:** Factors associated with underweight among adult prisoners with respiratory infection in Tigray region in 2013/2014, northern Ethiopia (*n* = 787)

Variables		Crude OR (95% CI)	*p*-value	Adjusted OR (95% CI)	*P*-value
Age	5 year increase	0.91 (0.86, 0.98)	0.007**	0.95 (0.87, 1.03)	0.207
Family support	No	1.00		1.00	
	Yes	0.58 (0.42, 0.79)	<0.001***	0.61 (0.43, 0.85)	0.004**
Marital status	Married	1.00		1.00	
	Single	1.45 (1.04, 2.01)	0.027*	0.87 (0.58, 1.31)	0.499
	Divorced/widowed	0.87 (0.42, 1.81)	0.708	0.50 (0.23, 1.09)	0.081
Educational status	No formal education	1.00			
	Has formal education	1.21 (0.84, 1.76)	0.313	0.93 (0.60, 1.44)	0.752
Occupation	Unemployed	1.00		1.00	
	Civil servant	0.56 (0.30, 1.03)	0.061	0.68 (0.35, 1.32)	0.252
	Farmer	0.51 (0.33, 0.8)	0.003**	0.59 (0.36, 0.98)	0.042*
	Student	1.13 (0.65, 1.95)	0.674	1.16 (0.65, 2.08)	0.614
	Other occupations	0.72 (0.40, 1.30)	0.270	0.83 (0.45, 1.54)	0.552
Duration since jailed	1 year increase	1.04 (0.99, 1.10)	0.131	1.07 (1.01, 1.14)	0.026*
Khat chewing	No	1.00			
	Yes	2.68 (1.65, 4.35)	<0.001***	2.08 (1.17, 3.70)	0.013*
Smoking	No	1.00		1.00	
	Yes	1.61 (1.12, 2.33)	0.011*	0.92 (0.58, 1.46)	0.727
History of previous jail	No	1.00		1.00	
	Yes	1.84 (1.21, 2.81)	0.005**	1.54 (0.99, 2.42)	0.058
HIV	Negative	1.00		1.00	
	Positive	1.51 (0.74, 3.08)	0.254	1.50 (0.70, 3.21)	0.302
Has TB	No	1.00		1.00	
	Yes	1.83 (0.88, 3.81)	0.107	1.44 (0.66, 3.12)	0.360

*signifies for *p*-value <0.05, **signifies for *p*-value < 0.01, and ***signifies <0.001

Moreover, compared to prisoners who were unemployed before being jailed, prisoners who were farmers had a 41% (OR = 0.59; 95% CI = 0.36, 0.98) lower risk of becoming underweight. For every 1 year additional incarceration in the prison setups, the odds of underweight increases by 7% (OR = 1.07; 95% CI = 1.01, 1.14). Additionally, those prisoners with a history of previous incarceration had a 54% excess risk, (OR = 1.54; 95% CI = 0.99, 2.42), of getting underweight compared to those who were jailed for their first time (Table 3).

Prisoners who had HIV (OR = 1.50; 95% CI = 0.70, 3.21) and TB (OR = 1.44; 95% CI = 0.66, 3.12) had 50% and 44% higher odds of being underweight, respectively. However, these differences were statistically insignificant (Table 3).

**Fig. 2:**
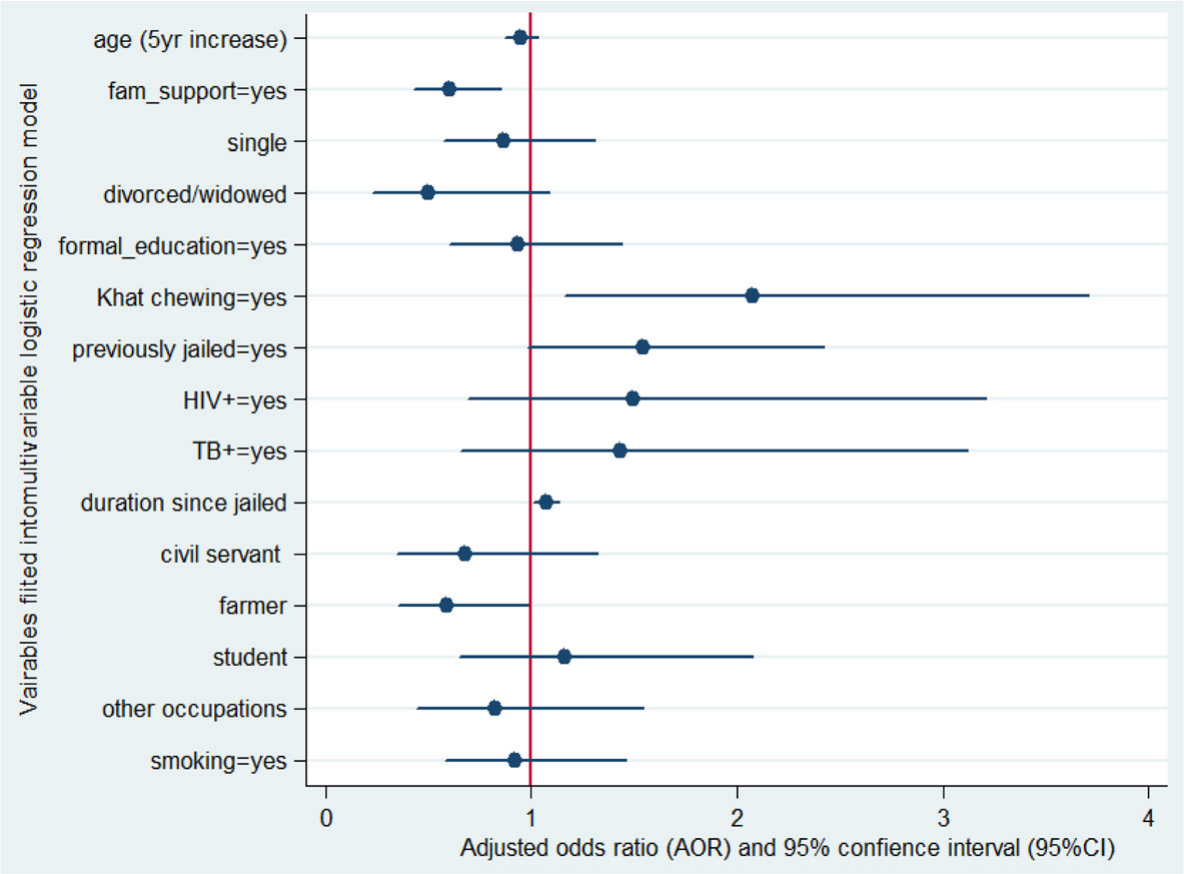
Visual plots of the adjusted odds ratios, along with 95% CI, for each of fitted variable in to the multivariable binary logistic model

Figure 2 shows the independent effect of each fitted variable represented by forest plots. The dot in each plot represents the estimate of the adjusted odds ratio and the width of each plot shows the 95% confidence interval of the corresponding adjusted odds ratio (Fig. 2).

## Discussion

In general, this study demonstrates that underweight affects one in every four of our sample population. Khat chewing, prolonged duration of incarceration and unemployment were significant determinants of underweight in the incarcerated population. Previous history of incarceration was also an important determinant with a borderline significance. Moreover, family support was a significant protective determinant of underweight among the prisoners.

One of the main goals of this study was to determine the burden of underweight among adult prisoners who had respiratory tract infections. We found that 25.2% of the adult prisoners were underweight, of which 35.8% were either severely or moderately underweight, which suggests that underweight is a significant public health problem among prisoners in Tigray region.

The high prevalence of underweight observed among the prisoners could be partly influenced by many factors. One of the important justifications comes from the clinical fact that respiratory infection by itself could lead to malnutrition [34] by mechanisms such as weight loss due to increased basal metabolic rate, poor appetite and metabolic losses of nutrients. However, malnutrition could also be a risk factor for increased risk of infections [35–37] and may be an important indicator of prognosis from infectious diseases of the respiratory system [38, 39]. Additionally, multiple other studies have also demonstrated that prisoners were suffering from several micronutrient deficiencies that could predispose them to severe forms of infections in addition to other health problems such as depression and mortality [9–11, 14, 19, 40–44]. In our study, 9% of the prisoners were either severely or moderately underweight. Hence, implementing therapeutic feeding program for such prisoners and general food distributions for the mildly underweight and normal prisoners could lead a sharp decline of underweight rates [45] and contribute to prevention of epidemics in such vulnerable population. Since our study was implemented using cross-sectional design, as part of our study’s shortcoming, we are unable to demonstrate whether the respiratory infections had caused underweight or the reverse. However, the study indicates that nutritional needs of prisoners should be optimized to ensure adequate nutritional status of prisoners.

Moreover, morbidity from respiratory infections of at least two weeks could also be associated with poor access to appropriate health care service, as reported in several studies [46–49]. This might be related to prolonged loss of appetite and loss of sleep. On the other hand, the high prevalence of underweight among the convicts could be due to low rations and quality of food served on top of severe prison related stress [50, 51]. Previous studies have also reported that prisoners faced a wide spectrum of nutritional problems [10–12, 14, 41, 52–54] and diets were not balanced [13, 54]. Nevertheless, it is important to note that our observed burden of underweight among the prisoners could be overestimated due to the fact that our study was based on prisoners who had respiratory/TB infection in which weight loss is a typical feature of such health problems.

One of the key determinants of underweight in the incarcerated population was chewing khat. Prisoners who chewed khat had a two-fold increased risk of becoming underweight. Sleep disturbance and loss of appetite associated with khat chewing could be the main explanations for the observed higher risk of underweight among khat chewing prisoners compared to their counterparts [55–58], rendering them susceptible to subsequent immunosuppression and repeated cycles of respiratory infections [35]. Such a deadly vicious cycle of interaction of undernutrition and infection, combined with possible deficiencies of several micronutrients, might further lead the prisoners to higher risk of severe morbidity and mortality.

Our study showed that there was a 41% lower risk of underweight for prisoners who were getting family support. Although we could not find literature linking family support and nutritional status of prisoners, it is a straight forward observation in that those who secured family support could be more likely to eat enough, achieve emotional and social support that could neutralize possible stress, and maintain a healthy life style such as personal hygiene. We assume that these health promoting conditions could finally lead them to lower risk of underweight compared to their counterparts.

Another important finding was that every one year additional imprisonment significantly translated to a 7% higher risk of underweight. This could imply that compared to prisoners who were imprisoned only for 1 year, those prisoners who were imprisoned for five years had approximately 35% higher risk of becoming underweight. Prior studies have also reported that longer duration of imprisonment was associated with significantly lower vitamin D level and lower values of serum proteins, mean corpuscular volume and haematocrit [12, 19, 54]. Within two years of incarceration, a 22 year old Nigerian detainee was found to develop severe underweight characterized by irreversible severe sensory and motor neuropathy of lower limbs and pulmonary TB [59]. Such severe underweight, also referred as “hunger disease”, was also reported to be associated with death [51]. The higher risk of becoming underweight with longer duration of imprisonment could probably be due to harsh living conditions that might arise from long term exposure to psychological stress, overcrowding, possible humiliation, hunger, morbidity, poor personal hygiene and sanitation [51]. In a study done elsewhere, longer duration of incarceration was also associated with risk of drug use [60]. To further explain how longer duration of incarceration relates with underweight, in our analysis, we constructed a statistical scenario that examined if individuals with strong family support who were employed prior to their incarceration have better nutritional status early in their incarceration. If so, this might justify that food provided by prison institutions is of poor nutritional value over time. However, the analysis of the interaction of family support, duration of incarceration and employment status does not support the constructed scenario. Therefore, further research should be done to disentangle how longer duration of incarceration influences nutritional status of prisoners. Nevertheless, we strongly recommend that nutrition interventions in prison setups should be approached in an interdisciplinary and holistic perspective considering the duration of confinement and other public health factors, including overcrowding and poor environmental sanitation, to effectively address nutritional problems and promote overall health of prisoners. Moreover, prospective research should also aim to identify the critical time that prisoners become at risk of underweight and develop other nutritional disorders in order to widen the window of intervention opportunity.

Moreover, we have observed 54% higher risk of underweight for prisoners who had history of previous incarceration with a borderline significance. Studies have shown that re-imprisonment was significantly determined by mental health, previous jail, younger age, drug and alcohol use [61–64]. Additionally, prisoners with previous history of incarceration had 40% more risk to have general medical problems [15] and poor clinical outcomes such as lower odds of HIV-1 suppression [65]. The findings of the abovementioned previous studies could also explain for the high burden of underweight observed in our study.

Unemployed prisoners were significantly associated with increased risk of underweight compared to farmers. Similarly, studies done in different settings have revealed that unemployment was associated with food insecurity, malnutrition and TB [66–69]. Poor economic access due to unemployment before incarceration could expose prisoners to risks of poor food quality, low food ration and other health related risk factors which may have led these prisoners to excess risk of underweight.

On the other hand, this study was able to demonstrate that TB, HIV sero-positivity and younger age were also associated with higher risk of underweight, but these associations were statistically insignificant. The absence of significant association between TB/HIV status and risk of underweight could be underestimated due to the small number of such clinical cases which might have neutralized the potentially pronounced reverse effects of these infections on the nutritional status of the prisoners.

This study has three important shortcomings which must be mentioned for cautious utilization of the results. First, this study was implemented among prisoners who had respiratory tract infections. Therefore, we assume that our findings may not be generalizable to all other prisoners in the sense that the level of underweight might have been overestimated due to the fact that weight loss is one of the typical features among individuals who are TB putative cases. Second, potential confounding variables of underweight such as mental health status were not adjusted in the multivariable analysis because it was the study’s exclusion criteria due to the assumption that the data may not be reliable enough if the data would have come by interviewing such cases. A notable consequence of this is that our findings and conclusions could have been affected by a residual confounding although we did not quantify the level of this confounding. Third, despite the detailed training given to the data collectors as well as supervisors, strict implementation of our study procedures and measurements of the variables to enhance the validity and precision of our study, our attempt of quantifying the dose, frequency and duration of smoking and khat chewing was not precise enough. Due to this limitation, we were forced to perform our analyses simply based on the status of smoking and khat chewing. This might have limited our understanding of the potential effects of such unhealthy practices on the outcome variable.

This study, covering the whole major prison sites in the regional state and the first of its kind from Ethiopia, enquires into the extent of the problem of underweight, which is mostly an overlooked public health problem in the marginalized prison population particularly in developing countries. Hence, this study extends our knowledge about underweight and its associated factors in prisons in the context of Ethiopia which may have an enormous implication in initiating possible nutritional interventions in such a vulnerable population.

## Conclusion

To conclude, the prevalence of underweight in the prison setups was high. Moderate and severe forms of underweight constitute more than one-third of the prevalent underweight cases. Absence of family support, khat chewing, longer duration of incarceration and unemployment were strong determinants of underweight. Previous history of incarceration and morbidities due to TB and HIV were also important factors worth considering, although the associations were statistically insignificant. We strongly recommend that political leaders, researchers and non-governmental organizations (NGOs) come together to integrate holistic nutritional interventions to address underweight and reform the environmental health conditions to address underweight and promote overall health status of prisoners. Implementing therapeutic feeding programs for severely or moderately underweight prisoners should be one priority component of any nutritional interventions in prison settings.

## Abbreviations

BMI: Body mass index
CI: Confidence interval
CM: Centimeter
FMoH: Federal Ministry of Health
GOF: Goodness of fit
HCT: HIV Counseling and Testing
HIV: Human Immunodeficiency Virus
ICPR: Institute for Criminal Policy Research
IQR: Inter Quartile Range
KG: Kilogram
NGOs: Non-governmental organizations
OR: Odds ratio
PTB: Pulmonary tuberculosis
TB: Tuberculosis
UDHR: Universal Declaration of Human Rights
VIF: Variance inflation factor
WHO: World Health Organization
WPB: World Prison Brief
